# High Sensitivity Refractometer Based on a Tapered-Single Mode-No Core-Single Mode Fiber Structure

**DOI:** 10.3390/s19071722

**Published:** 2019-04-10

**Authors:** Wenlei Yang, Shuo Zhang, Tao Geng, Le Li, Guoan Li, Yijia Gong, Kai Zhang, Chengguo Tong, Chunlian Lu, Weimin Sun, Libo Yuan

**Affiliations:** 1 Key Lab of In-fiber Integrated Optics, Ministry Education of China, Harbin Engineering University, Harbin 150001, China; yangwenlei@hrbeu.edu.cn (W.Y.); an0814@hrbeu.edu.cn (G.L.); gongyijia0322@163.com (Y.G.); zhangkai@hrbeu.edu.cn (K.Z.); luchunlian@hrbeu.edu.cn (C.L.); sunweimin@hrbeu.edu.cn (W.S.); 2 Centre for Composite Materials and Structures, Key Laboratory of Science and Technology for National Defence, Harbin Institute of Technology (HIT), Harbin 150080, China; lile_19851125@163.com; 3 School of Electronic Engineering and Automation, Guilin University of Electronic Technology, Guilin 541004, China; lbyuan@vip.sina.com (L.Y.); chengguotong73@163.com (C.T.)

**Keywords:** fiber optics sensor, refractive index sensor, no core fiber

## Abstract

We have proposed a novel tapered-single mode-no core-single mode (TSNS) fiber refractometer based on multimode interference. The TSNS structure exhibits a high contrast ratio (>15 dB) and a uniform interference fringe. The influence of different lengths and diameters of the TSNS on the refractive index unit (RIU) sensitivity was investigated. The experimental investigations indicated a maximum sensitivity of 1517.28 nm/RIU for a refractive index of 1.417 and low-temperature sensitivity (<10 pm/°C). The experimental and simulation results are also in good agreement.

## 1. Introduction

Optical fiber refractive index sensors have several advantages such as minimization, stability, fast response, and so on. They can be utilized as the basis of many photonic devices, such as biosensors and chemical sensing systems because of their unique advantages [1,2]. Various optical fiber structures have been used to fabricate different refractive index sensors, for example, the single mode-multimode-single mode (SMS) structure based on the multimode interference (MMI), Bragg grating, and long-period fiber grating [3,4,5]. Among them, SMS refractometers based on the MMI have attracted extensive attention because of their simple structure, good repeatability, and low cost of preparation [6,7,8]. Similar to the role of the multimode fiber (MMF) in the MMI sensor, a piece of no core fiber (NCF), which is made of pure quartz, also can be directly used to serve as the MMI area and sensing head. Moreover, the NCF can leak light into the surrounding environment more easily and has a lower thermo-optic coefficient so that the temperature cross-sensitivity is avoided when measuring the refractive index. Some researchers have demonstrated that the NCF can be prepared for many refractive index sensors [9,10]. Chen et al. fabricated a self-temperature-compensative refractometer using a piece of NCF [11]. Zhou et al. used a section of NCF spliced with a single-mode fiber (SMF) as a sensing head and light was reflected back by the gold film to fabricate the refractive index sensor [12]. Thus, interferometers based on the single mode-no core-single mode (SNS) fiber structure have great potential as refractive index sensors.

Most refractometers based on the MMI rely on interference patterns for refractive index measurements. Previous reports have shown that the refractive index or geometry modulation of the MMI structure increases the refractive index sensitivity of the sensor [13,14]. However, the interference patterns formed by multiple mode coupling deteriorates with the increase in the refractive index so as to reduce the spectrum quality for sensing signal demodulation [15,16]. These problems result in difficulties in selecting reference wavelengths. Therefore, it is necessary to obtain stable interference patterns while ensuring high sensing sensitivity.

In previous work, SNS or SMS structures exhibit stable interference fringes before reducing the diameter of the fiber. They use taper technology just to get a stronger evanescent field. In this paper, a novel TSNS refractometer was prepared and studied in detail. TSNS structure is made of a standard diameter SNS structure using taper technology. No mode interference affects the original SNS structure. As the length of the TSNS increases, the optical path difference increases, and visible interference fringes appear in the transmission spectrum. Because of the smaller diameter of tapered optical fiber, the number of modes participating in the interference decreases which effectively improves the visibility of interference fringes, and the refractive index sensitivity of the TSNS is effectively improved. The maximum refractive index sensitivity reaches up to 1517.28 nm/RIU for a refractive index of 1.417. Under the same external refractive index, the refractive index response of the new TSNS structure is higher than that of the traditional MMI sensor [17,18,19]. The effect of the diameter and length of the sensor on the spectral and refractive index response was theoretically and experimentally investigated and the temperature sensing characteristics were experimentally determined. The test results show that multiple experimental samples have similar temperature responses (about 10 pm/°C).

## 2. Principle of Operation and Theoretical Analysis

**Figure 1:**
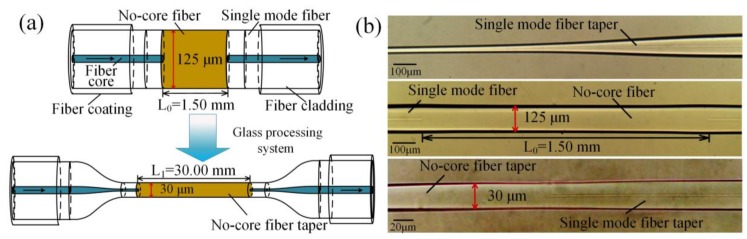
(**a**) Preparation process, geometry parameters of the SNS and TSNS structure. (**b**) microscopic images of the TSNS structure.

The SNS fiber structure is composed of a section of NCF sandwiched between two SMFs. A precision preparation device and special fusion splice parameters were used to construct the SNS fiber structure [20]. The TSNS fiber structures were obtained after the SNS structure passed through a glass processing system (GPX-3000 glass processing system, Vytran Corp). The preparation process and geometry parameters of the TSNS are shown in Figure 1. After repeated optimization of the preparation techniques, we have achieved a better appearance of the TSNS fiber structure. The NCF is fully machined and the diameter of the TSNS is reduced from 125 μm to 30 μm. The microscopic images of the SNS and TSNS structure are also shown in Figure 1. The much smaller diameter of the tapered NCF than the standard fiber should be able to control the number of high-order eigenmodes when several high-order eigenmodes are stimulated. In addition, according to fiber evanescent field theory, with the decrease of the diameter of optical fibers, the evanescent field of optical fibers becomes stronger. Therefore, light in the taper region is more sensitive when the external refractive index changes [21]. The stronger evanescent field brought by the smaller diameter improves the sensing response of the TSNS refractometer.

In the case of ideal splicing and perfect alignment, it can be assumed that excited high-order modes (LP_0m_) and the fundamental mode (LP_01_) propagated in the TSNS structure. The interference fringe is formed by the interactions between the LP_0m_ and the fundamental mode (LP_01_) when light travels from the input SMF to the output SMF. The light field distribution $E\left(r,0\right)$ at the first splicing point is expressed as:

(1) $$E\left(r,0\right)=\sum_{m=1}^{M}\eta_{m}\psi_{m}\left(r\right)$$

Here, the field distribution of LP_0m_ is $\psi_{m}\left(r\right)$ when light enters the TSNS structure. The refractive index of the fiber core and cladding and core diameter determine $\psi_{m}\left(r\right)$. The excitation coefficient for each mode ($\eta_{m}$) can be expressed as:

(2) $$\eta_{m}=\frac{\int_{0}^{\infty }E\left(r,0\right)\psi_{m}\left(r\right)rdr}{\int_{0}^{\infty }\psi_{m}{\left(r\right)}^{2}rdr}$$

Therefore, the distribution of the light field in the direction of light propagation *z* (*z = L*) can be expressed as:

(3) $$E\left(r,L\right)=\sum_{m=1}^{M}\eta_{m}\psi_{m}\left(r\right)\exp\left(jk_{m}L\right)$$

where *k_m_* is the propagation constant of the excited eigenmodes in the TSNS fiber structure. Thus, at the output terminal, the normalized energy distribution *P*_s_(*z*) can be expressed using the overlap integral:

(4) $$P_{s}\left(z\right)=\frac{{\left|\int_{0}^{\infty }E\left(r,z\right)E_{01}\left(r\right)rdr\right|}^{2}}{\int_{0}^{\infty }{\left|E\left(r,z\right)\right|}^{2}rdr\int_{0}^{\infty }{\left|E_{01}\left(r\right)\right|}^{2}rdr}$$

The field distribution $\psi_{m}\left(r\right)$ in Equation (3) and *z* (*z = L*) in Equation (4) both affect the distribution of the output light field and then affect the output transmission spectrum. The coupling coefficient $\eta_{m}$, which varies with the change of the refractive index, leads to changes in the final energy distribution for different refractive indices [19].

**Figure 2:**
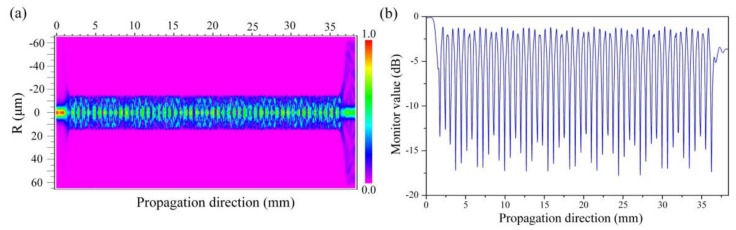
(**a**) Amplitude distribution of the propagation field of the TSNS at a wavelength of 1550 nm using the 3D-FD-BPM. (**b**) Energy ratio at the center of the model (x = 0 μm) at the resonance wavelength.

The TSNS structure is assumed to be isotropic and circularly symmetric. These assumptions improve the simulation efficiency and reduce the difficulty of the simulation. Based on the above theoretical analysis and assumptions, the transmission characteristics can be simulated using the 3-dimensional finite difference beam propagating method (3D-FD-BPM). The light propagation along the TSNS structure (*L*_1_ = 30 mm, *D* = 30 μm) is shown in Figure 2a. It is evident that the high-order mode is excited, and the energy is continuously exchanged in the TSNS structure to form a stable interference fringe and the contrast ratio is also more than 15 dB in Figure 2b. These features clearly differ from the transmission characteristics of the traditional SNS structure [6]. In the simulation, the parameters of the SMF and NCF were chosen as: $n_{cl}^{SMF}$= 1.4468, $n_{co}^{SMF}$ = 1.4520 and $n^{NCF}$ = 1.4500. The external medium was a transitional boundary condition (TBC) (*n* = 1). The mesh sizes of the X and Y directions were 0.1 μm and the mesh size of the transmission direction (*z*) was 0.5 μm.

**Figure 3:**
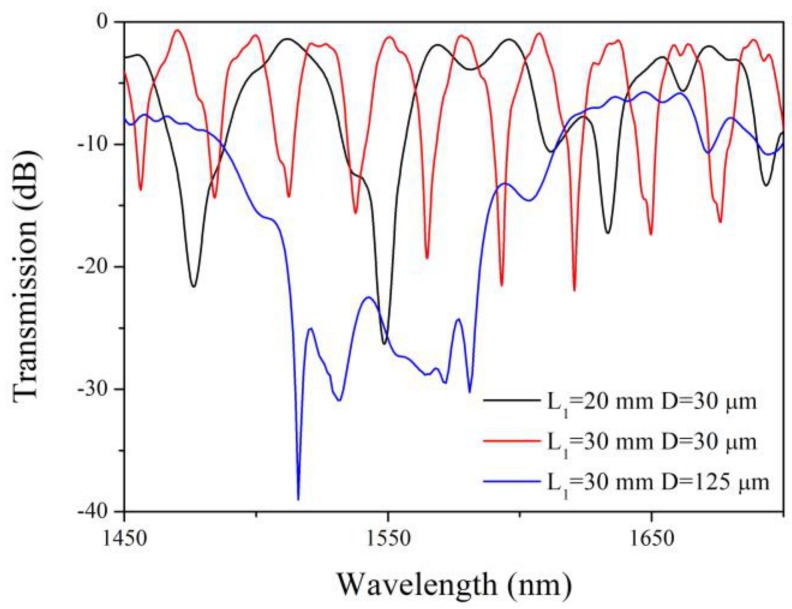
Simulated transmission spectrum of the three types of TSNSs.

Figure 3 shows the simulation transmission spectrum of the three types of TSNSs by wavelength scan (the scanning step is 0.2 nm). In the case of the fixed length, the change in taper diameter has a significant effect on the transmission spectrum. The TSNS structure with a standard diameter (*D* = 125 μm) has a higher insertion loss and a more disorganized transmission spectrum. When the diameter of the NCF is reduced to 30 μm, the tapered NCF is still a multimode waveguide but the number of modes is less than that of the NCF of 125 μm. In the case of a fixed diameter, the spacing between adjacent interference peaks gradually decreases with increasing interference length. A clear interference fringe is obtained as the red curve in Figure 3.

**Figure 4:**
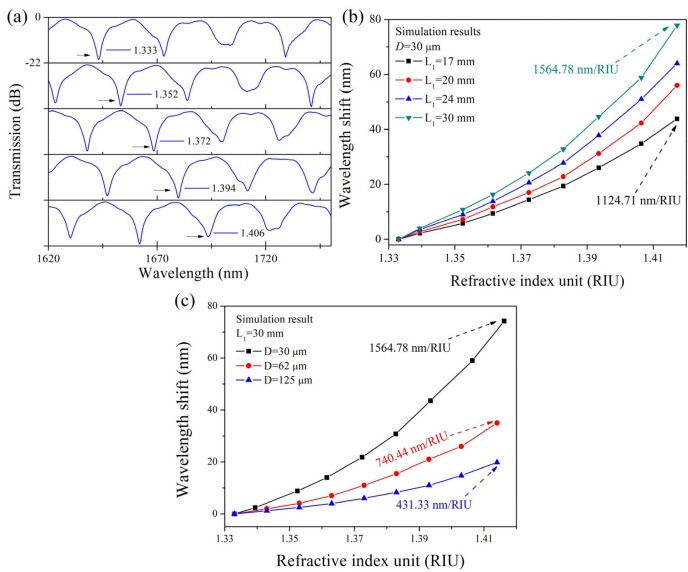
(**a**) Spectral shift at different refractive indices (*L*_1_ = 30 mm, *D* = 30 μm). Refractive index response of TSNS structure in the simulation (all *y*-axis ranges are 0 to −22 dB). (**b**) Different lengths. (**c**) Different diameters.

The refractive index changes should be manifested in the shift of the interference pattern. We used simulation models to simulate the refractive index response of the TSNS structure. The spectral shifts at different refractive indices are shown in Figure 4a. As the external refractive index increases, the interference fringe of the TSNS structures exhibits a significant red shift. Most interference peaks still maintain a high contrast ratio as the refractive index increases. Figure 4b,c show the external refractive index response (the external refractive index range is 1.333 to 1.417) of the TSNS structures with different lengths and diameters. The red shift of the interference fringes becomes increasingly apparent with the increase in the external refractive index. The sensitivity of the interference fringe greatly increases and reaches a maximum of 1564.78 nm/RIU. A comparison of all cases indicates that the refractometer with a shorter TSNS exhibits lower sensitivities and the change of the fiber diameter is more noticeable than the effect of the length on the sensitivity.

## 3. Spectral Characteristics and Refractive Index Experiment

When passing through the length *L*_1_ of the TSNS fiber, the phase difference between the LP_01_ and LP_0m_ is defined as:

(5) $$\theta^{m}=\frac{2\pi \Delta n_{1,m}^{eff}L_{1}}{\lambda }$$

where $\Delta n_{1,m}^{eff}$ is the effective refractive index difference between LP_01_ and LP_0m_ and λ is the Free Spectral Range (FSR). From Equation (5), the wavelength interval of the two interference minima for the interference fringes generated in the TSNS can be approximated as [22]:

(6) $$\Delta \lambda \approx \frac{\lambda^{2}}{\Delta n_{1,m}^{eff}L_{1}}$$

As shown in Equation (6), the wavelength interval of the two interference minima will decrease with the increase in the interferometer length *L*_1_. Further, the wavelength of the *m*-order interference minima will shift with the change in the external refractive index as shown in Equation (7):

(7) $$\Delta \lambda_{m}=\frac{2\left(\Delta n_{1,\;m}^{eff}+\Delta n\right)L}{2m+1}-\frac{2\left(\Delta n_{1,\;m}^{eff}\right)L}{2m+1}=\frac{2\left(\Delta n\right)L}{2m+1}$$

Δn is the change in the effective refractive index of the TSNS fiber structure as a result of the change in the surrounding refractive index. As the external refractive index increases, the right side of the equation is positive and $\Delta \lambda_{m}$ shifts towards a longer wavelength (red-shift) [23].

**Figure 5:**
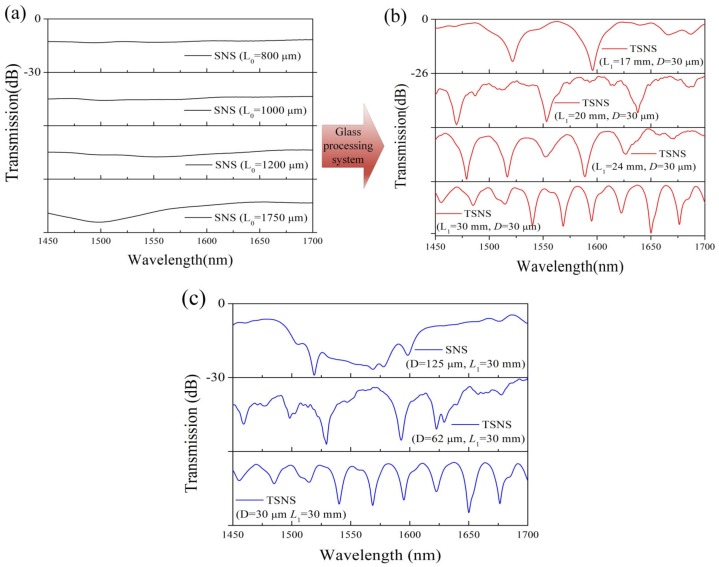
(**a**) Transmitted spectrum of the original SNS fiber structures. Transmission spectrum of the TSNS for different parameters (all *y*-axis ranges are 0 to −30 dB). (**b**) Different lengths (all *y*-axis ranges are 0 to −26 dB). (**c**) Different diameters (all *y*-axis ranges are 0 to −30 dB).

According to the discussion all above, several experiments were implemented using different lengths of TSNS structures. We fabricated four kinds of TSNS structures (17, 20, 24, and 30 mm) using SNS structures with different lengths. Transmission spectral characteristics of SNS structures and the four kinds of TSNS are shown in Figure 5.

Figure 5a shows that the insertion loss of the original SNS structure increases slightly with increasing length. These SNS structures do not have obvious interference patterns. Figure 5b shows that the transmission spectrum of the TSNS structures with different lengths. The density of the interference fringes increases with the increase in the length of the TSNS structure. The contrast ratio of the interference fringe is high, and the interference minima have better uniformity. As shown in Figure 5c, the TSNS structure with a smaller diameter is significantly superior to the traditional SNS structure with regard to the clarity of interference fringes.

**Figure 6:**
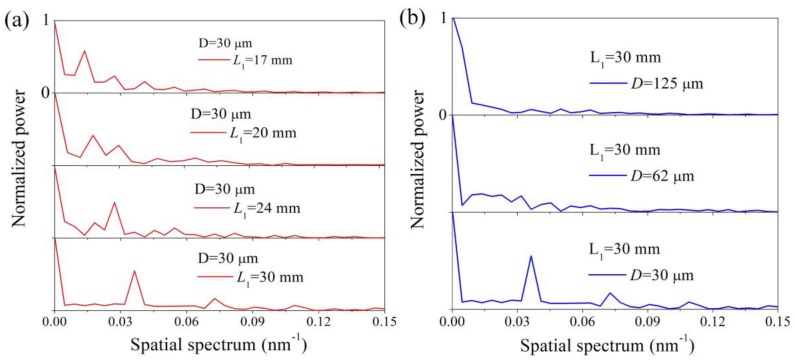
Spatial spectra of the TSNS fiber structures (all *y*-axis ranges are 0 to 1). (**a**) Different lengths. (**b**) Different diameters.

In order to verify the regularities of the spectral characteristics of the different TSNS structures, the transmission spectra in Figure 5 are transformed into the spatial frequency using the Fourier transform, as shown in Figure 6.

The relationship between the spatial frequency and the interferometer length was given as [24,25]:

(8) $$\xi =\frac{1}{\lambda_{0}{}^{2}}\Delta m^{eff}L_{1}$$

(9) $$\Delta m^{eff}=\Delta n_{m}^{{}^{eff}}-\lambda_{0}\frac{\partial }{\partial \lambda }\Delta n_{m}^{{}^{eff}}$$

where *L*_1_ is the length of the TSNS, $\lambda_{0}$ is the center wavelength of a Taylor expansion, ξ is the spatial frequency, and $\Delta m^{eff}$ is the differential modal group index. When $\Delta m^{eff}$(or *L*_1_) is fixed, the relationship between *L*_1_ (or $\Delta m^{eff}$) and the spatial frequency ξ is positively related. There are indeed one dominantly excited eigenmode and some faintly excited higher-order eigenmodes in each spatial spectrum. Assuming that $\Delta m^{eff}$ is fixed, the spatial frequency of the dominant eigenmode increases with the increase in the interference length as shown in Figure 6a. In Figure 6b, several cladding modes were excited and participated in the interference. As the diameter of the NCF decreases, the number of modes participating in the interference decreases and the power of the dominant high-order mode gradually increases. When the diameter of the NCF is reduced to 30 μm, a dominant cladding mode is significantly stimulated and forms clear interference fringes with the fundamental mode.

**Figure 7:**
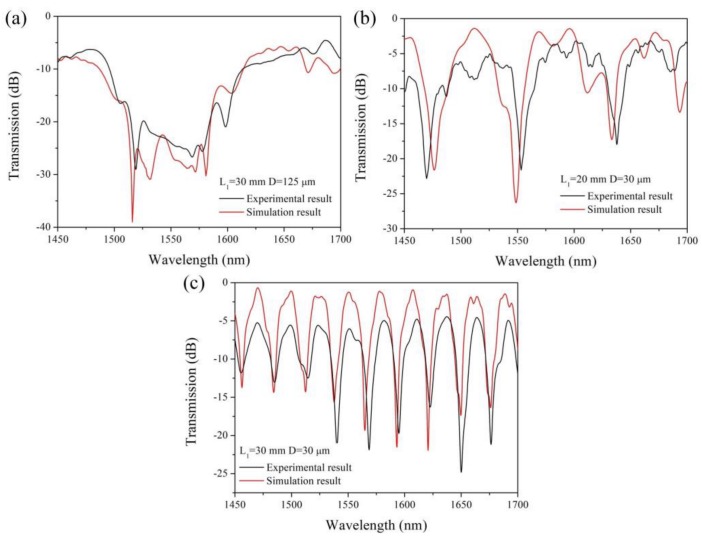
Simulated and experimental results of TSNS (**a**) *L*_1_ = 30 mm, *D* = 125 μm (**b**) *L*_1_ = 20 mm, *D* = 30 μm (**c**) *L*_1_ = 30 mm, *D* = 30 μm.

The comparison of the simulated and experimental results of the spectral characteristics is shown in Figure 7 (in air, n = 1). The wavelength scan results show a good agreement between the simulated and experimental results with regard to the interference fringe. The small difference in the additional insertion loss and wavelength between the experiment and simulation are mainly attributed to the approximation method used in the simulation and small errors in the preparation process.

**Figure 8:**
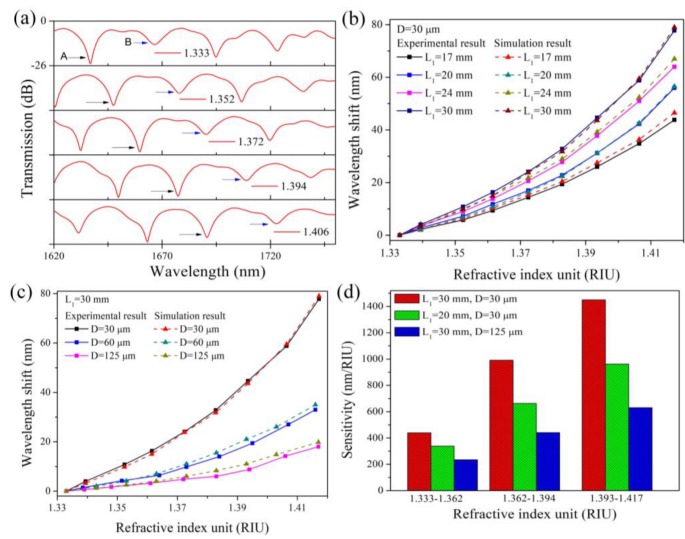
(**a**) Transmission spectra for different refractive indices. Refractive index response of TSNS structure in the experiment (all *y*-axis ranges are 0 to −26 dB). (**b**) Different lengths. (**c**) Different diameters. (**d**) The average sensitivities in three refractive index regions.

The external refractive index response of the TSNS fiber structure was verified experimentally. The test was carried out at room temperature (25 °C). The refractive index matching fluids (1.333–1.417) were obtained using different ratios of glycerol-aqueous solution. During the experiment, the TSNS structure was immersed into the different refractive index matching solutions. Light was injected into the TSNS fiber structure using a super-continuum (SC) light source and the transmission spectrum of the sensor was monitored at the output using a spectrum analyzer (Agilent AQ6317B, spectral range 600–1750 nm). The transmission spectra of the TSNS refractometer (*L*_1_ = 30 mm, *D* = 30 μm) with different external refractive indices are shown in Figure 8a. As shown in the Figure 8a, two reference peaks A and B with different contrast ratio (reference peaks A and B are pointed out in the figure by black and blue arrows) in the wavelength range of 1620–1750 nm. With the increase of the refractive index, it is clear in the measurement range that the two reference peaks are red-shifted together. In this pair of reference peaks, the interference peak B with a lower contrast ratio (the blue arrow) is used as a marker, and we trace the deeper reference peak A at 1636.8 nm (the black arrow) to obtain the wavelength shift characteristic. As the external refractive index increases, the interference pattern undergoes a large red shift. The refractive index responses are obtained by polynomial fitting: y_1_ = 6953.72x^2^ − 18,189.56x + 11,890.69; the refractive index characteristics have a good fit in the fitted region (R^2^ = 0.99).

The experimental wavelength shift behaviors of the different lengths and diameters TSNS refractometers are shown in Figure 8b,c. It is evident that the maximum refractive index sensitivity increases significantly with the increase in the interference length and decrease in the diameter. The rate of increase is higher at a higher refractive index than at a lower refractive index in all cases. The TSNS (L_1_ = 30 mm, D = 30 μm) provides the best result among these cases and reaches 1517.28 nm/RIU at a refractive index of 1.417. The above experimental results are consistent with our theoretical simulation. For ease of practical applications, the three refractive index ranges of the constant sensitivity are given: 1.333–1.362, 1.362–1.394, and 1.394–1.417. The average sensitivities in the three regions are 439.11 nm/RIU, 991.18 nm/RIU, and 1450.31 nm/RIU, respectively.

Table 1 compares previous MMI sensors with our work. Our work is highly sensitive in comparison. In addition, our work has stronger interference fringe visibility than ref. [18] in actual measurements and avoids the dangerous chemical corrosion of ref. [19].

## 4. Temperature Characteristics and Results Discussion

**Figure 9:**
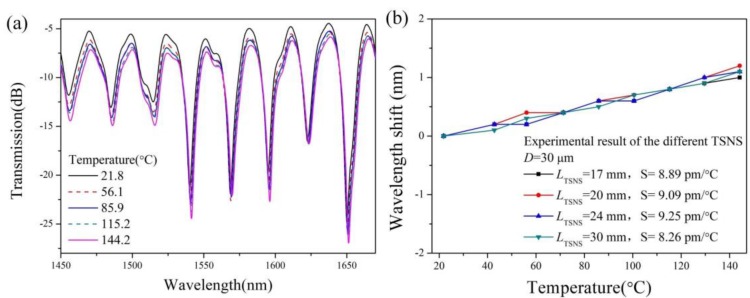
(**a**) Transmission spectral response at different temperatures. (**b**) Linear-fitting function of wavelength-temperature.

The cross-sensitivity of the temperature has a very important influence in terms of the refractive index measurement. An insensitive temperature response is desired for the refractive index test, within a certain temperature range. Therefore, we studied the temperature characteristics of the TSNS structure using four samples. The samples were placed on a thermostatic furnace. After many experiments, the temperature sensing characteristics of the TSNS structure were obtained as shown in Figure 9a. With increasing temperatures (21.8–144.2 °C), the wavelength of the TSNS refractometer only undergoes a slight red shift (<10 pm/°C). Figure 9b shows the linear regression analysis of the temperature characteristics. The sensitivities of the four samples in the test range are 8.26 pm/°C, 9.25 pm/°C, 9.09 pm/°C, and 8.89 pm/°C and the R^2^ values are all 0.99. As shown in Figure 9b, better temperature robustness was maintained in the 4 samples with a red shift (only about 1 nm) occurring in the tested temperature range. The low-temperature sensitivity is attributed to the NCF prepared from pure silica because it has a similar thermo-optic coefficient of the pure silica between the LP_01_ and LP_0m_ [26].

## 5. Conclusions

In this study, a new type of high sensitivity refractometer was fabricated and studied using welding and tapering techniques. A simulation model based on the 3D-FD-BPM is used to theoretically study the response to the external refractive index. The results confirm that a smaller diameter of the tapered area effectively improves the refractive index sensitivity and has a strong influence on the spectrum. A longer TSNS will result in more intensive interference fringes and higher refractive index sensitivity. The experimental investigations show that the refractive index sensitivity reaches a maximum of 1517.28 nm/RIU for a refractive index of 1.417. The experimental results are in good agreement with the simulation results. The temperature sensitivity of the samples is highly consistent. Stronger temperature robustness more effectively avoids the cross-sensitivity of the temperature during the refractive index measurement. Due to the simple preparation method and the stable temperature characteristics, the TSNS fiber refractometer has high application potentials in bio-sensing and chemical sensing systems when coating special materials.

## Figures and Tables

**Table 1:** Comparison of Sensitivity for MMI refractometer.

Sensitivity of RIU	Refractive Index Range	Configuration	References
−110.5 nm/RIU	1.341 to 1.3676	A SMS fiber Michelson interferometer	[7]
259 nm/RIU	1.333 to 1.381	A single-mode-no core-single-mode (SNS) fiber structure	[10]
327 nm/RIU	1.33 to 1.38	Reflective smf-small diameter no core fiber structure	[17]
1900 nm/RIU (at a RI of 1.44)	1.33 to 1.44	A tapered, multimode fiber interference	[18]
1815 nm/RIU	1.342 to 1.437	A SMS fiber structure using multimode fiber core and SMF	[19]
1517.28 nm/RIU	1.333 to 1.417	TSNS fiber structure	Our work
